# Genetic diversity of aflatoxin-producing *Aspergillus flavus* isolated from selected groundnut growing agro-ecological zones of Uganda

**DOI:** 10.1186/s12866-020-01924-2

**Published:** 2020-08-14

**Authors:** Amos Acur, Renée S. Arias, Steven Odongo, Samuel Tuhaise, Joseph Ssekandi, John Adriko, Dennis Muhanguzi, Stephen Buah, Andrew Kiggundu

**Affiliations:** 1 National Agricultural Research Laboratories, P.O. Box 7065, Kampala, Uganda; 2 National Peanut Research Laboratories, P.O. Box 509, 1011 Forrester Drive, S.E, Dawson, GA 39842 USA; 3 College of Veterinary Medicine Animal Resources and Biosecurity, Makerere University, P.O. Box 7062, Kampala, Uganda

**Keywords:** Mycotoxins, Secondary metabolites, *Aspergillus* species and agro-ecological zone

## Abstract

**Background:**

Groundnut pre- and post-harvest contamination is commonly caused by fungi from the Genus *Aspergillus*. *Aspergillus flavus* is the most important of these fungi. It belongs to section *Flavi*; a group consisting of aflatoxigenic (*A. flavus*, *A. parasiticus* and *A. nomius*) and non-aflatoxigenic (*A. oryzae*, *A. sojae* and *A. tamarii*) fungi. Aflatoxins are food-borne toxic secondary metabolites of *Aspergillus* species associated with severe hepatic carcinoma and children stuntedness. Despite the well-known public health significance of aflatoxicosis, there is a paucity of information about the prevalence, genetic diversity and population structure of *A. flavus* in different groundnut growing agro-ecological zones of Uganda. This cross-sectional study was therefore conducted to fill this knowledge gap.

**Results:**

The overall pre- and post-harvest groundnut contamination rates with *A. flavus* were 30.0 and 39.2% respectively. Pre- and post-harvest groundnut contamination rates with *A. flavus* across AEZs were; 2.5 and 50.0%; (West Nile), 55.0 and 35.0% (Lake Kyoga Basin) and 32.5 and 32.5% (Lake Victoria Basin) respectively. There was no significant difference (χ^2^ = 2, *p* = 0.157) in overall pre- and post-harvest groundnut contamination rates with *A. flavus* and similarly no significant difference (χ^2^ = 6, *p* = 0.199) was observed in the pre- and post-harvest contamination of groundnut with *A. flavus* across the three AEZs. The LKB had the highest incidence of aflatoxin-producing *Aspergillus* isolates while WN had no single *Aspergillus* isolate with aflatoxin-producing potential. *Aspergillus* isolates from the pre-harvest groundnut samples had insignificantly higher incidence of aflatoxin production (χ^2^ = 2.667, *p* = 0.264) than those from the post-harvest groundnut samples. Overall, *A. flavus* isolates exhibited moderate level (92%, *p* = 0.02) of genetic diversity across the three AEZs and low level (8%, *p* = 0.05) of genetic diversity within the individual AEZs. There was a weak positive correlation (*r* = 0.1241, *p* = 0.045) between genetic distance and geographic distance among *A. flavus* populations in the LKB, suggesting that genetic differentiation in the LKB population might be associated to geographic distance. A very weak positive correlation existed between genetic variation and geographic location in the entire study area (*r* = 0.01, *p* = 0.471), LVB farming system (*r* = 0.0141, *p* = 0.412) and WN farming system (*r* = 0.02, *p* = 0.478). Hierarchical clustering using the unweighted pair group method with arithmetic means (UPGMA) revealed two main clusters of genetically similar *A. flavus* isolates.

**Conclusions:**

These findings provide evidence that genetic differentiation in *A. flavus* populations is independent of geographic distance. This information can be valuable in the development of a suitable biocontrol management strategy of aflatoxin-producing *A. flavus.*

## Background

Groundnut (*Arachis hypogaea* L*.*), is a major legume grown on approximately 25 million hectares of the semi-arid tropical and sub-tropical regions of the world between latitudes 40 ^°^N and 40 ^°^S. The global annual production is estimated at 36 million tons [1]. Groundnut is the second most important legume in Uganda after the common bean (*Phaseolus vulgaris*) [2]. The consumption of groundnut in Uganda is in either of the following forms: roasted seeds, groundnut stew, groundnut paste and sometimes raw seed cake is used as animal feed.

Despite its importance as a food and cash crop, groundnut faces production and export constraints from mycotoxin accumulation that results from contamination by *Aspergillus* species favoured by the tropical climate experienced in Uganda [3]. Mycotoxins are secondary metabolites produced during fungal metabolism in response to environmental stress. The toxins are secreted in defence, virulence or cell signalling [4]. These secondary metabolites are stable compounds that cannot be degraded by any ordinary cooking temperature or food processing procedures [5–7]. When these compounds are ingested, they cause problems to both human and livestock health in the form of acute illness, chronic illness, instant death or immunosuppression among others [5]. In most cases, the effects of mycotoxins are manifested much later after exposure [6].

Human and livestock exposure to mycotoxins in developing countries result from over-reliance on a single staple food crop which is normally grown only once a year [8]. Therefore, this food commodity is kept much longer under storage in order to prolong its availability awaiting new harvest in the succeeding year. Since most storage facilities in developing countries are improvised structures, a great proportion of the stored crop produce get contaminated by *Aspergillus* species, resulting into changes in taste, colour, odour and nutritional value of food and feeds [9].

The economic losses due to *Aspergillus* contamination may reach 100% when the presence of aflatoxins beyond acceptable levels results in produce rejection [10]. Acute aflatoxicosis is often as a result of subject exposure to high doses of aflatoxins resulting into instant death, whereas chronic aflatoxicosis is due to exposure to sub-lethal doses over a long period of time [11]. Chronic aflatoxicosis results in liver cancer, immune suppression and teratogenicity among other complications [11]. This problem is a common occurrence in developing countries like Uganda where farmers have inadequate food storage facilities and poor food handling practices [12]. In addition, in developing countries, no strict regulatory measures exist against high levels of aflatoxins in food and feedstuffs, leading to frequent episodes of aflatoxicosis and often death in humans [13].

The ability of fungal species to produce aflatoxins is strain-specific and it is controlled by aflatoxin biosynthesis gene cluster, consisting of *aflR*, *aflS*, *aflP*, *aflQ*, *aflD*, *aflM* and *aflO* genes [14]. Sequence variability in this aflatoxin biosynthesis gene cluster has always been useful in deducing diversity in aflatoxigenic *Aspergillus flavus* species [15]. At the moment, no scientific reports have been published about the genetic diversity of indigenous *A. flavus* population in Uganda. The objective of the present study was therefore to assess the contamination rates of groundnut with major *Aspergillus* species and to examine the genetic diversity of indigenous *A. flavus* isolated from groundnut in six representative districts within the agro-ecological zones (AEZs) of Uganda using InDel markers located within the aflatoxin biosynthesis gene cluster.

## Results

The dominant fungal species contaminating groundnut in Uganda is not well understood due to the complexity in the underlying causes including geographical and genotypic factors. Two hundred and forty groundnut samples were collected from the three AEZs of Uganda for fungal isolation and characterisation. In total, 231 *Aspergillus spp.* isolates were identified from the groundnut samples collected (Table 1). The isolates comprised of *A. flavus*, *A. parasiticus* and *Aspergillus* section *Nigri*. Typically, more than one *Aspergillus* species were found co-existing on 70.0% (168/240) of the total groundnut samples collected and on 61.0% (73/120) of the post-harvest groundnut samples. *Aspergillus flavus* was the most abundant, both as S- and L-strains, whereas *A. parasiticus* was the least abundant species observed (Table 1). The three *Aspergillus* species were distributed throughout the AEZs surveyed with LKB having the highest abundance of *A. flavus* and WN with the least (Table 1). *Aspergillus* section *Nigri* was most abundant in the LVB and least abundant in WN (Table 1). *Aspergillus parasiticus* was least abundant in equal proportions across the three AEZs (Table 1). However, the abundance and distribution of each species never differed significantly across AEZs (*p* = 0.165).

**Table 1:** *Aspergillus* species and strains identified by AEZs

Agro-ecological zone	*A. flavus* (S strain) *n* = 88	*A. flavus* (L strain) *n* = 46	*A. parasiticus n* = 3	*Aspergillus* section *Nigri* *n* = 94
West Nile	10 (11.36%)	2 (4.35%)	1 (33.33%)	18 (19.15%)
L. Victoria basin	36 (40.91%)	14 (30.43%)	1 (33.33%)	46 (48.94%)
L. Kyoga basin	42 (47.73%)	30 (65.22%)	1 (33.33%)	30 (31.91%)
**Total** **(*N*** **= 231)**	**88 (38.10%)**	**46 (19.91%)**	**3 (1.30%)**	**94 (40.69%)**

Overall, 34.6% (83/240) of the groundnut samples collected had *Aspergillus*. The post-harvest groundnut samples were more contaminated than the pre-harvest groundnut samples with the contamination frequencies of 39.2 and 30.0% respectively. Generally, Lake Kyoga basin mixed-farming system had the highest number of groundnut samples contaminated with *Aspergillus* and WN farming system had the lowest number of groundnut samples contaminated with *Aspergillus* (Table 2). At the pre-harvest level, LKB had the highest *Aspergillus* contamination frequency whereas the WN farming system had the lowest (Table 2). The post-harvest contamination frequency was highest in WN farming system while the LVB farming system had the lowest (Table 2). Although the contamination proportions (both pre- and post-harvest) varied in the three AEZs (Table 2), the Pearson’s Chi square test revealed no significant difference in *Aspergillus* contamination among the three AEZs (χ^2^ = 6, *p* = 0.199).

**Table 2:** Proportions of groundnut samples contaminated by *Aspergillus* at pre-and post-harvest

Agro-ecological zone	Pre-harvest contamination (n = 40)	Post-harvest contamination (*n* = 40)	Overall contamination (*n* = 80)
West Nile	1 (2.50%)	20 (50.00%)	21 (26.25%)
L. Victoria basin	13 (32.50%)	13 (32.50%)	26 (32.50%)
L. Kyoga basin	22 (55.00%)	14 (35.00%)	36 (45.00%)
**Total (*N*** **= 120)**	**36 (30.00%)**	**47 (39.17%)**	**83 (34.58%)**

Examination of the aflatoxin production capacity of selected *Aspergillus* isolates showed that LKB had the highest incidence of isolates with aflatoxin-producing potential whereas WN had no single isolate capable of producing aflatoxins (Table 3). The incidence of aflatoxin production was higher among *Aspergillus* isolated from the pre-harvest groundnut samples of LKB origin and from the post-harvest groundnut samples of LVB origin (Table 3). In general, the incidence of aflatoxin production by *Aspergillus* isolates from the pre-harvest groundnut samples was insignificantly higher (χ^2^ = 2.667, *p* = 0.264) than that of *Aspergillus* isolates from the post-harvest groundnut samples across AEZs (Table 3).

**Table 3:** Aflatoxin production potential of selected *Aspergillus* section *Flavi* isolates from three agro-ecological zones at pre- and post-harvest stages

AEZ	Isolate	Sample type	Sample status	Total aflatoxins (ppb)	Incidence (%)
**Lake Kyoga Basin**	SS-1468-LKB	Pre-harvest	Positive	169	83.33%
*n* = 6	SS-1513-LKB	Pre-harvest	Positive	364	
	SS-1607-LKB	Pre-harvest	Positive	121	
	SS-1463-LKB	Pre-harvest	Positive	841	
	SS-1485-LKB	Pre-harvest	Positive	1429	
	SS-1517-LKB	Pre-harvest	Negative	0	16.67%
n = 6	SS-1546-LKB	Post-harvest	Positive	0.42	16.67%
	LS-1478-LKB	Post-harvest	Negative	0	83.33%
	LS-1603-LKB	Post-harvest	Negative	0	
	LS 1605-LKB	Post-harvest	Negative	0	
	LS-1554-LKB	Post-harvest	Negative	0	
	SS-1473-LKB	Post-harvest	Negative	0	
**Lake Victoria Basin**	SS-1525-LVB	Pre-harvest	Positive	598	16.67%
n = 6	SS-1453-LVB	Pre-harvest	Negative	0	83.33%
	SS-1524-LVB	Pre-harvest	Negative	0	
	SS-1520-LVB	Pre-harvest	Negative	0	
	SS-1549-LVB	Pre-harvest	Negative	0	
	SS-1458-LVB	Pre-harvest	Negative	0	
n = 6	SS-1629-LVB	Post-harvest	Positive	9	50.00%
	SS-1526-LVB	Post-harvest	Positive	648	
	SS-1527-LVB	Post-harvest	Positive	175	
	SS-1453-LVB	Post-harvest	Negative	0	50.00%
	SS-1536-LVB	Post-harvest	Negative	0	
	SS-1626-LVB	Post-harvest	Negative	0	
**West Nile** n = 6	LS-1509-WN	Pre-harvest	Negative	0	100%
	LS-1454-WN	Pre-harvest	Negative	0	
	SS-1511-WN	Pre-harvest	Negative	0	
	SS-1486-WN	Pre-harvest	Negative	0	
	SS-1496-WN	Pre-harvest	Negative	0	
	SS-1627-WN	Pre-harvest	Negative	0	
n = 6	SS-1568-WN	Post-harvest	Negative	0	100%
	SS-1536-WN	Post-harvest	Negative	0	
	SS-1456-WN	Post-harvest	Negative	0	
	SS-1493-WN	Post-harvest	Negative	0	
	SS-1457-WN	Post-harvest	Negative	0	
	SS-1487-WN	Post-harvest	Negative	0	
***N*** **= 36**					

**Fig. 1:**
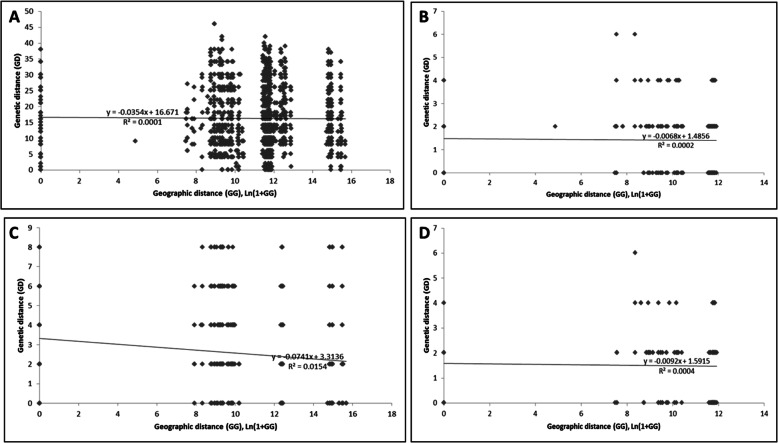
Isolation by distance plots. Genetic distance plotted as a function of geographic distance for the entire study area (*p* = 0.471, *r* = 0.01) (**a**), the Lake Victoria basin farming system (*p* = 0.412, *r* = 0.0141) (**b**), the Lake Kyoga basin farming system (*p* = 0.045, *r* = 0.1241) (**c**) and the West Nile farming system (*p* = 0.474*, r* = 0.02) (**d**). The bold line represents the line of best fit to the plotted data and *r* values represent the correlation between geographic and genetic distances matrices assessed using the Mantel test

Out of the 137 *Aspergillus* section *Flavi* isolates (*A. flavus* and *A. parasiticus*), 96 representative isolates were fingerprinted using 25 InDel-primed PCR markers at 65 loci of the aflatoxin biosynthesis gene cluster. After the clonal correction, only 67 out of 96 isolates had unique allele scores generated from 16 out of the 25 InDel markers and at 24 loci out of the total 65 loci of the gene cluster genotyped. Fingerprint data of these 67 isolates were then used in the subsequent statistical analyses. Based on the Mantel tests of correlation between matrices of geographic and genetic distances, there was no significant isolation by distance for the entire study area (*r* = 0.01, *p* = 0.471) (Fig. 1a). When each AEZ was examined separately, isolation by distance between the *A. flavus* populations was detected in the LKB, with a weak positive correlation (*r* = 0.1241, *p* = 0.045) (Fig. 1c) between the geographical distance and genetic distance. Similarly, there was also a weak genetic divergence of 0.0154 in the LKB due to geographic distance (Fig. 1c). The rest of the AEZs had insignificant positive correlations between the geographic distances and genetic distances (Fig. 1b and d).

**Fig. 2:**
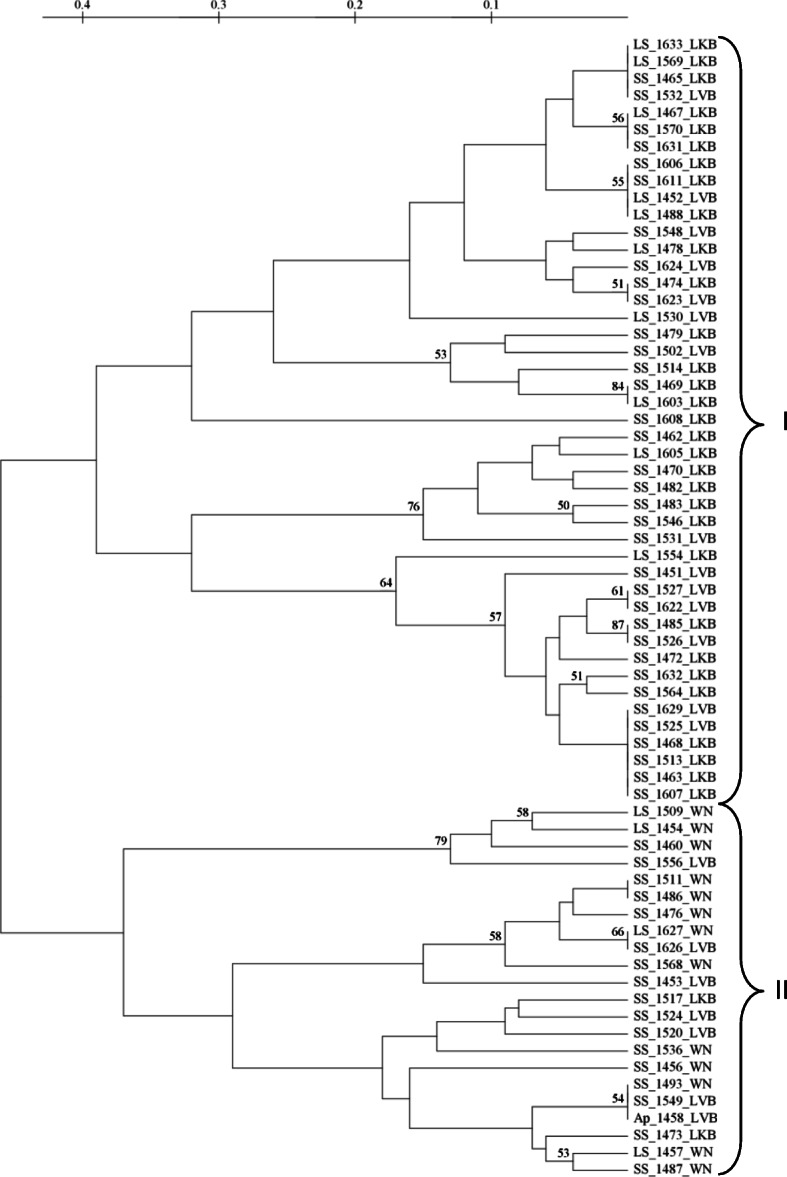
Dendrogram of genetic relationships among 67 *Aspergillus* isolates. The result from a fingerprint data based on InDel markers used in genotyping 67 isolates collected from three agro-ecological zones using the UPGMA algorithm and the genetic distances at 1000 replications. The initials SS and LS represent S and L strains of *A. flavus* respectively while AP represents *A. parasiticus*. Clusters I and II comprise of aflatoxigenic and non-aflatoxigenic isolates respectively

The dendrogram generated using UPGMA algorithm revealed two major clusters that corresponded to the amplification patterns within the aflatoxin biosynthesis gene cluster of each isolate displayed in a capillary electrophoregram. From this electrophoregram, isolates with the most amplified portions within their aflatoxin biosynthesis gene cluster were in cluster I while isolates with the least amplified portions within the aflatoxin biosynthesis gene cluster were in cluster II (Fig. 2). The result from total aflatoxins analysis using an ELISA kit on the presentative isolates picked from each AEZ confirmed that isolates in cluster I were aflatoxigenic while those in cluster II were non-aflatoxigenic.

Analysis of molecular variance reflected that variation within populations and variation between regions (AEZs) were significant and accounted for genetic variation of 92 and 8% respectively (Table 4). Therefore, most of the observed genetic differentiation was due to variation within populations.

**Table 4:** AMOVA summary that describes the proportion of genetic variance in *Aspergillus* section *Flavi* isolates at different hierarchical levels

Source of variation	df	SS	MS	Est. var	%	*P-*value
Among regions	2	3.418	1.709	0.089	8%	0.05
Among populations	1	0.17	0.17	0	0%	0.927
Within populations	63	65.487	1.039	1.039	92%	0.02
**Total**	66	69.075		1.128	100%	

## Discussion

Considerable achievements have been made in controlling aflatoxins contamination of agricultural products following the discovery that aflatoxins are extremely toxic secondary metabolites to both humans and livestock [16, 17]. This study sought to elucidate the diversity of *Aspergillus* species contaminating groundnuts collected at pre- and post-harvest stages from three different agroecologies of Uganda. Due to seasonal variations in the different agroecologies, it was impossible to collect all samples from the study areas at the same time. In addition to sample collection constraints, we identified *Aspergillus* species and strains using stereoscopic microscopy, which is still considered a standard practice for microbial identification, but were unable to confirm the species identities using molecular tools.

In this study, *Aspergillus* section *Flavi* and *Aspergillus* section *Nigri* were the most abundant *Aspergillus* species encountered in groundnut samples*.* This finding concurs with that from a study done by Rathod & Naikade [18]. Most of the *Aspergillus* species in section *Nigri* are of great importance in the industrial manufacture of amylases, lipases, citric acid and gluconic acid [19]. However, they cause food deterioration with subsequent production of mycotoxins; ochratoxin A (OTA), ochratoxin B, fumonisin B2, fumonisin B4, and secalonic acids, A, D, F as the major natural products toxic to humans and animals [20]. Ochratoxin A has been reported to be a nephrotoxic compound causing renal cancer [21]. Groundnut contamination with *A. flavus* and some species from *Aspergillus* section *Nigri* is also known for lowering the germination ability of groundnut seeds under storage [22], and the longer the storage duration, the higher the frequency of contamination by *Aspergillus* species [23]. Thus, reduction in seed quality due to *Aspergillus* contamination results in poor seed germination ability, low productivity and hence food insecurity.

In general, presence of *A. flavus* as the most abundant and distributed fungal contaminant of groundnut is consistent with previous reports by Klich [12]; Bhatnagar et al. [10]; and Cotty & Jaime [24] . Groundnut contamination by *Aspergillus* species originates at the pre-harvest stage as was previously noted by Ligia et al. [25] in their study. This is because *Aspergillus* species are well adapted in the soil as conidia, hyphae and sclerotia, which are in direct contact with groundnut pods [26, 27]. Other abiotic factors like drought stress, a common experience in the LKB mixed farming system could be responsible for high susceptibility of groundnut at pre-harvest stage [17, 18, 28].

One of the interesting findings from this study was the lower *Aspergillus* contamination rates in WN farming system. This observation could be due to a higher altitude in WN farming system that is associated with relatively lower temperatures and relative humidity compared to the other AEZs. Cropping system and climate could be responsible for *Aspergillus* species distribution pattern and abundance within these AEZs that are far apart and with different climatic conditions as was noted by Horn & Dorner [29]. No significant statistical difference existed between pre-harvest and post-harvest contamination levels of groundnut with *Aspergillus* species across AEZs. This could be due to common pre- and post-harvest handling methods employed as previously noted by Dube & Maphosa [30].

When *A. flavus* isolates from the pre- and post-harvest groundnut samples were tested and incidences for aflatoxins production compared, no significant difference in aflatoxin-production ability was observed across AEZs. This is in agreement with Torres et al. [31], confirming that both the pre- and post-harvest groundnut can habour aflatoxin-producing *Aspergillus* provided there are suitable environmental conditions for *Aspergillus* growth and aflatoxins production.

Analysis of molecular variance (AMOVA) revealed that variation within populations was most responsible for genetic differentiation as compared to variation among populations and variation among regions (AEZs). Genetic variations in *A. flavus* within populations could be due to different cropping practices employed in each field from which samples were taken [30–32]. Furthermore, the AMOVA results suggest the existence of low genetic differentiation among regions (AEZs). This could be evidence of gene flow between the different AEZs as a result of human activities like trade and transportation of *Aspergillus*-contaminated groundnuts between AEZs. Another reason could be based on differential competition strategies employed by different genotypes of *Aspergillus* section *Flavi* to survive in diverse environments. For example, an isolate which is highly competitive during sporulation may exhibit dominance over a wide geographical region during multiple reproductive cycles although it is a poor competitor during crop infection. All these are likely to have important impacts on the *A. flavus* population structure.

On the other hand, genotypes that are dominant within the host tissues are better adapted to surviving in an AEZ having harsh environmental conditions that do not favour mechanisms for sporulation, dispersal and secondary infections [33]. Insignificant isolation by distance in the entire study area implies that there was gene flow leading to low genetic differentiation among AEZs while significant isolation by distance observed in the Lake Kyoga basin farming system is an indication that nearby populations in this AEZ were genetically more similar than expected by chance, and genetic differences increases linearly with geographic distances. This particular AEZ is semi-arid and therefore most strains of *A. flavus* in this region are adapted to living within the plant host tissues as a means of adapting to unfavourable environmental conditions. They, therefore, tend to remain localized in particular geographical locations within the AEZ since sporulation and dispersal that could have limited genetic isolation by distance are limited by harsh environmental conditions [34].

The variations between regions (AEZs) shown by the AMOVA results could have been as a result of differences in the period that *Aspergillus* was in association with groundnut in each AEZ. Temporal differences in the stages at which *Aspergillus* is in association with groundnuts, either at pre- or post-harvest could also be responsible for the observed population differentiation among regions. Each AEZ has its unique colonization stages for groundnut-associated *Aspergillus* and genetic adaptations undergone by the *Aspergillus* for its host plant differ with the stage of growth of the plant. This means AEZs may also differ in temporal stages of association of *Aspergillus* with groundnuts right from the initial colonization till the development of correlated spatial genetic structure over time. Two major clades are due to the insertion/deletion in the aflatoxin biosynthesis gene cluster of the fingerprinted *A. flavus* isolates. Since this gene cluster has a set of conserved genes that regulate the biosynthesis and secretion of aflatoxins by the fungus, any alteration in their sequences either by insertion or deletion can lead to non-aflatoxigenicity [35, 36].

## Conclusion

*Aspergillus* section *Flavi* and *Aspergillus* section *Nigri* are the most abundant species contaminating groundnuts in Uganda. They co-exist in groundnuts posing serious health risks to both humans and animals. Since contamination starts at the pre-harvest stage due to drought stress, early planting should be emphasized so that there is enough rain to take the plants through their growth and development. In case of unexpected drought, simple irrigation technologies should be adopted to meet the water demands of the plants, especially during pod development stage.

The genetic diversity of *A. flavus* in various AEZs provides a gene pool of potential value for application in biocontrol. This can be exploited to reduce the prevalence of aflatoxigenic fungi in the environment through competitive exclusion mechanism. From the results of genetic diversity analyses of *A. flavus* populations in the three selected AEZs, any non-aflatoxigenic strain of *A. flavus* can be selected as a biocontrol strain since there is no significant population differentiation by geographical distance. This strategy has been proven to be successful and it is already being applied in many countries that intensively produce groundnut, like the US [17]. Furthermore, the identified aflatoxigenic strains of *A*. *flavus* can be used to screen for resistance to *Aspergillus* colonization and subsequent aflatoxins production in different crop cultivars during a crop breeding programme.

Future studies could investigate the role of spatial and temporal variations on the genetic structure of *A.flavus* populations associated with groundnuts and also validate recently reported molecular markers by Hussain et al. [36] for detecting afltoxigenic strains of *A. flavus* on groundnuts collected from various environments.

## Methods

### Sample collection

**Fig. 3:**
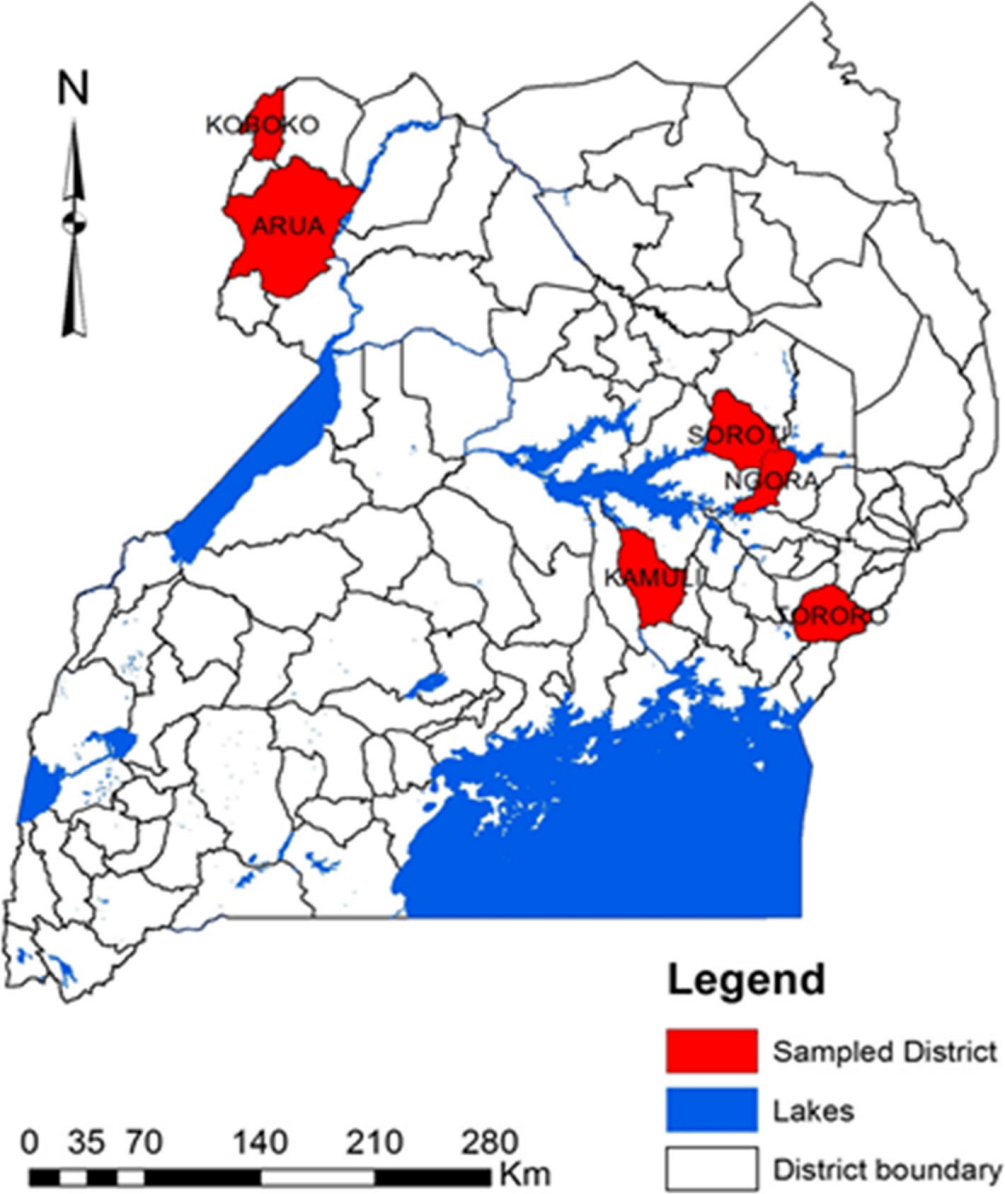
Location and zonal context of selected agro-ecological zones. Six districts were sampled from the entire study area, two districts from each agro-ecological zone to obtain groundnuts for *Aspergillus* isolation. Source: Uganda Bureau of Statistics [37]

There were three levels to the sampling design: (i) AEZs, (ii) districts within AEZ, and (iii) individual fields within the districts (Fig. 3). Not all sites were sampled during the same cropping season. Agro-ecological zones were selected to represent a range of agro-ecologies in Uganda known for groundnut production according to the Uganda Bureau of Statistics report, 2014. Two districts from each groundnut-growing AEZ were randomly selected and surveyed. The selected major groundnut-growing AEZs of Uganda included: West Nile farming system (WN), high altitude districts (districts of Arua and Koboko), Lake Kyoga basin mixed farming system (LKB), low altitude districts (districts of Soroti and Ngora) and Lake Victoria basin farming system (LVB), mid-altitude districts (districts of Tororo and Kamuli) (Fig. 3).

Households and groundnut fields in selected AEZs were surveyed for sample collection. Forty groundnut samples, consisting of 20 field samples and 20 storage samples, were collected from each selected district. For field collection, a quadrat measuring 1 m × 1 m was thrown randomly at five different sampling points and any distance ≥10 m between sampling points was considered. Three groundnut stands were pulled from each sampling point and handpicked on the same day of sampling. Extra care was taken to sort out pods that were damaged by soil fauna and later clean pods packed in a paper bag. The groundnut pods were sun-dried for a week, disinfected using a 0.5% (v/v) sodium hypochlorite solution, hand shelled and stored at 4 °C until fungal isolation according to Mutegi et al. [38].

Sub-samples of storage shelled groundnuts were collected randomly from each bag or container from the top, middle and bottom using a sampling probe and later mixed to form a composite sample. About 250 g of the composite sample were drawn and packed in a sterile paper bag for fungal isolation. Unshelled groundnuts were taken only once from each storage bag or container and packed for laboratory analysis following the method of Ndungu et al. [39].

### Isolation of *Aspergillus* species from groundnut seeds

*Aspergillus* species were isolated at National Peanut Research Laboratory, Dawson, Georgia, USA. A selective growth medium, modified dichloran Rose Bengal (MDRB) was used for isolation of *Aspergillus* section *Flavi* [29]. The MDRB medium is composed of 10 g/L dextrose, 2.5 g/L peptone, 1.0 g/L di-potassium phosphate (KH_2_PO_4_)_,_ 0.5 g/L magnesium sulphate heptahydrate (MgSO_4_.7H_2_O), 0.5 g/L yeast extract, 20 g/L agar, 0.5 mL of 0.05% (w/v) Rose Bengal stock solution in acetone, adjusted to 1 L with distilled water and later modified with 0.8 mg/L dichloran. After sterilization, 30 mg/L streptomycin and 0.15 mg/L tetracycline were added to the medium. Twenty seeds per sample were put into separate sterile 50 mL falcon tubes and 15 mL sterile distilled water added to each. The seeds were washed by shaking in a pulverizing machine, KLECO (Visalia, California, USA) for 2 min [40]. Thereafter, 50 μL of each of the suspensions was separately spread plated onto MDRB medium [41], followed by incubation at 37 °C for 3 days. Colonies of *Aspergillus* present per sample were counted, and contamination incidence (%) for each AEZ was deduced by counting the number of sample plates having ≥ 25 colonies of aflatoxin-producing *Aspergillus* species in each AEZ, (*n* = 120) according to Horn & Dorner [42]. In a biosafety cabinet, a stereo microscope and a flame sterilized needle were used to isolate conidia from the colonies of interest in each culture plate. Direct isolation of pure colonies of *A. flavus* and *A. parasiticus* from the MDRB culture plates was based on the size of conidiophores, vesicles, conidia and on the colour of conidial heads [43]. Colonies of *A. flavus* appear yellow-green to grey-green while those of *A. parasiticus* are dark green according to Christensen [43]. The fully grown *A. flavus* colonies are of two strains, the S strain and the L strain. The S strain is characterised by numerous sclerotia < 400 μm in diameter whereas the L strain produces fewer sclerotia > 400 μm in diameter [44]. Using a stereo microscope, the conidia from a single conidiophore were then picked and transferred onto freshly prepared plates of MDRB medium. In order to obtain a single colony from the picked conidia, streaking was done by successively turning the media plate in a right-angle manner in an attempt to adequately disperse the individual conidium at the extreme end of the streak. After three days of incubation at 37 °C, hyphal tips from single colonies were picked using a flamed scalpel and transferred into Czapek Dox agar (OXOID Ltd., Hampshire, England) slants for identification and storage.

### *Aspergillus* species and strain identification

Twelve-day old pure cultures of *Aspergillus* grown on Czapek Dox agar at 30 °C were morphologically characterized based on the distinguishing features for each morphotype as described above. Different species and strains were identified in accordance with Diba et al. [45], and comparison to reference cultures in the collection at National Peanut Research Laboratory, Dawson, GA, USA. Each isolate was derived from a sample and in some cases, two or more isolates would be derived from a single sample on condition that the colony characteristics showed different morphotypes; L- or S- strain (*A. flavus*) or *A. parasiticus*.

### Quantification of total aflatoxin from *A. flavus* mycelia

**Fig. 4:**
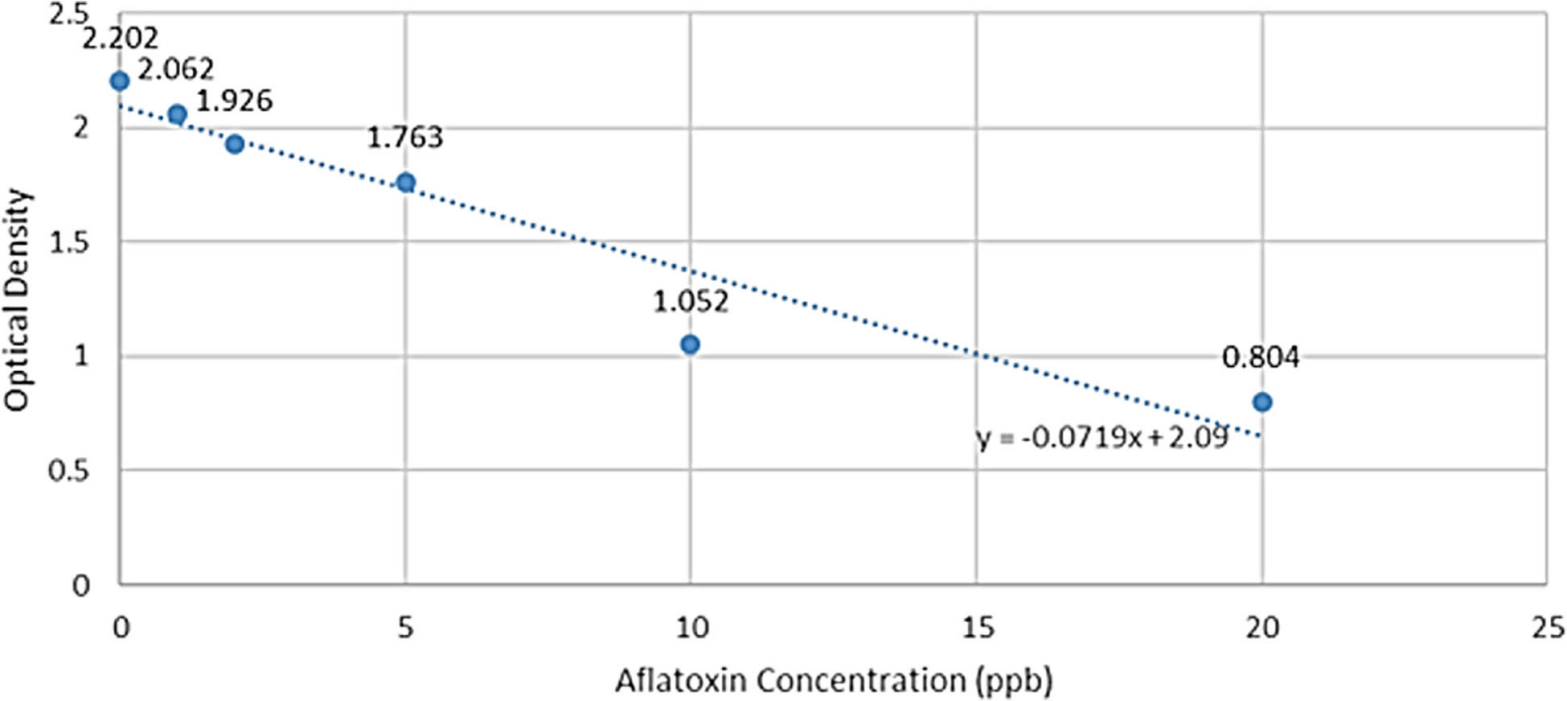
Standard curve for determination of aflatoxins concentration. The equation of the line was used to calculate the corresponding concentrations of aflatoxins in each sample based on the absorbance readings

This was done using the MaxSignal Total Aflatoxin ELISA Test kit (Bioo Scientific Corporation, Austin, Texas, USA). Representative *A. flavus* isolates consisting of 18 each from pre-harvest and from the post-harvest groundnut samples were randomly picked from each AEZ for total aflatoxin quantification. Each of the isolate was grown on potato dextrose agar at 37 °C for 7 days. Mycelia were extracted and ground for 3 min in a 3 ml extraction solution (70% v/v, methanol/water) using a sterile mortar and pestle. The ground samples were allowed to settle and the top layer of the extract filtered through a Whatman #1 filter paper. The resultant filtrate was treated with the kit’s reagents following manufacturer’s instructions. A microwell reader fitted with a 450 nm filter was used to read the optical density of the reaction mixture in each microwell. A standard curve was constructed using ELISA absorbance readings of the total aflatoxin standards (0, 0.02, 0.06, 0.2, 0.6 and 1.5 ng/mL) to determine aflatoxin concentrations in ppb. Absorbance readings and concentrations of the standard solutions were entered into a Microsoft Excel 2016 spreadsheet, and a standard curve was generated (Fig. 4). From this standard curve, concentrations of aflatoxins in corresponding samples were calculated using the equation of the line; y = − 0.0719x + 2.09 (y = optical density and x = aflatoxins concentration).

### Genomic DNA extraction and quantification

Genomic DNA from the *Aspergillus flavus* isolates was extracted at National Peanut Research Laboratory, Georgia, USA, using Qiagen DNeasy Plant kit (QIAGEN, Hilden, Germany). Sterile disposable plastic loops were used to harvest 3 loopful of spores from the culture slants and loaded into each sample tube. Following the manufacturer’s instructions, a 500 μl clear lysate was pipetted into a 2 ml eppendorf tube and later loaded into a QIAcube robot (QIAGEN, Hilden, Germany). The concentration of the eluted DNA was determined using a Nanodrop ND 2000 spectrophotometer (Thermo Fisher Scientific, Wilmington, DE, USA).

### Genotyping of *Aspergillus flavus* isolates

Primers that were previously developed by Faustinelli et al. [40] to detect insertions-deletions (InDels) within the aflatoxin-biosynthesis cluster of *Aspergillus* were used in this study for the genetic fingerprinting of the *Aspergillus* isolates (Table 5). Since deletions/insertions in this gene cluster are associated with aflatoxin production [35], clustering of isolates were deduced from the shared deletions/insertions patterns that correspond to aflatoxigenicity or non-aflatoxigenicity.

**Table 5:** Primer pairs that were used in the amplification of the aflatoxin biosynthesis gene [40]

Marker	Forward 5′ → 3’	Reverse 5′ → 3’	Amplicon size (bp)
AFLC01	CCGACCTCACGACGCATTAT	CCGGCTAGCTTCAACAGACG	140–370
AFLC02	GGTTGGCGGATTGAGAGGTA	GGAGATCAGCCGAGAAGACA	100–296
AFLC03	TCCGCCGAGAGCCATAATAG	GGATGCTGACACCTCGATAG	120–160
AFLC07	GTCAGCAAGAGGAGCCTTCA	GGTCACGGAGATCCTCCATA	159–404
AFLC08	CGCCAGCACGGAGATCGAAT	CGTCTCCTCAGGCGGTCTAT	224–399
AFLC12	CGCAAGGAGCTCGACCAATA	TTCAGCTCAGCGACGAGAGT	241–360
AFLC13	TCGGTTCAATGCTCGAACAC	TCCAACCTTCGGCCTAGTCT	140–410
AFLC15	GCTCTACAGGCTGATTCAAG	TCGACAGTCCGACAATATGC	204–370
AFLC16	ATCGCAGCGGAAGCTTGGAA	AGTCTCGGACTCCGGTGACA	145–410
AFLC17	GCACAACTCGTACAGCTATC	TCTAAGTGCGAGGCAACGAA	125–390
AFLC18	GGCAGCCAGACCAAGGAATA	CCTTCTCGTAGCCGCTCATC	130–400
AFLC19	ACAGGACCGCACGGATCAAT	AGGAGCGGATGTCGAAGTCT	260–491
AFLC20	GCCTAGCGCTCCATTCTCAG	CCATCGTATCCGGCTCTATC	120–370
AFLC21	TACCTTACTCCGCTAAGCAG	GCGGTCACCTACCAATGAAT	150–368
AFLC22	TTCGCAGGAGTGTAGCCAAG	GTTGGAACACGCTCCATAGG	120–371
AFLC24	GAACGAGATAACGGCTGCAT	ATCAATCCACGGACCGTTGT	100–430

The forward primers were tailed with a 5′-CAGTTTTCCCAGTCACGAC-3′ sequence and labelled with 6-carboxyfluorescein (6-FAM). The reverse primers were tailed with 5′-GTTT-3′ sequence to promote non-template adenylation [46]. Amplifications were performed using 10 ng of DNA and Titanium Taq polymerase (Clontech) in 5 μl reactions as described by Arias et al. [47]. The labelled PCR amplicons were analyzed using an ABI 3730XL DNA analyzer and data were processed by GeneMapper v 4.0 (Applied Biosystems, Foster City, California, USA).

### Data analysis

The GenStat Discovery Edition 14 (2002) for windows (VSN International Ltd., Rothamsted Experimental Station, UK) software was used for data analysis. The Chi-square test and One–Way analysis of variance (ANOVA) were used to compare the frequencies of groundnut contamination with *Aspergillus* species and to determine the relative abundance of isolated *Aspergillus* species and strains at pre- and post-harvest stages respectively. Allele sizes observed as relative fluorescence units from the electropherogram were converted to binary data, where the presence of an amplicon of any size was scored as ‘1’ whereas its absence was scored as ‘0’. The fingerprint data for all the samples were corrected for clonality by removing isolates sharing the same InDel profiles at all loci from the data. Analyses like population pair-wise Fst values and allele frequencies were performed on isolates’ alleles data in order to establish whether the sample size for each AEZ is adequate to be used in subsequent statistical analyses. After these, our analyses were restricted to unique isolates identified from the fingerprint data and to AEZs for which *A. flavus* isolates were available. Analysis of molecular variance was carried out using the program Arlequin version 3.5 for estimation of variance components and partition of the within and among population variance. In addition, isolation by distance (IBD) was assessed using the Mantel test that plots the genetic distance against geographic distance (log-transformed) for the entire study area and within districts using the program GenAIEx 6.5b. In both analyses, significance was assessed by conducting 999 permutations. During these analyses, sixteen InDel loci were used to identify genetic structure in *A. flavus* populations. Allele frequencies were calculated for *A. flavus* within each AEZ and geo-referenced. Allele frequencies were standardized by the lowest value and natural log-transformed while retaining zero values. Relationships among the isolates based on InDel data were determined through unweighted pair group method with arithmetic mean (UPGMA) with 1000 bootstrap replications [16], using TREECON for Windows version 1.3 b.

## Data Availability

The datasets generated during and/or analysed during the current study are available from the corresponding author on reasonable request.

## Abbreviations

AEZs: Agro-ecological zones
AMOVA: Analysis of molecular variance
ANOVA: Analysis of variance
DNA: Deoxyribonucleic acid
InDel: Insertion /deletion
MDRB: Modified dichloran Rose Bengal
PCoA: Principal Co-ordinate analysis
PCR: Polymerase Chain Reaction
ppb: Parts per billion
RNA: Ribonucleic acid
RNAi: Ribonucleic acid interference
UBOS: Uganda Bureau of Statistics
